# The effect of sex on vestibular schwannoma incidence varies across the lifespan and modifies associations with race/ethnicity

**DOI:** 10.21203/rs.3.rs-7645100/v1

**Published:** 2025-09-29

**Authors:** Kajal V. Parmar, Mackenzie Price, Syed M. Adil, Julia R. Benedetti, Carol Kruchko, Kyle M. Walsh, Quinn T. Ostrom

**Affiliations:** 1 Department of Neurosurgery, Duke University School of Medicine, Durham, NC, USA; 2 Central Brain Tumor Registry of the United States, Hinsdale, IL, USA; 3 The Preston Robert Tisch Brain Tumor Center, Duke University School of Medicine, Durham, NC, USA

**Keywords:** Vestibular schwannoma, Acoustic neuroma, African Americans, Risk factors, Incidence

## Abstract

**Purpose:**

Vestibular schwannoma (VS) is a benign tumor of the eighth cranial nerve with incidence that differs by age, sex, and race/ethnicity. Prior research has not characterized how the contributions of sex and race/ethnicity to VS risk may interact or vary by age. We sought to examine the joint contributions of age, sex, and race/ethnicity to VS risk using nationally-representative data.

**Methods:**

Diagnoses of non-malignant, intracranial VS were extracted from the Central Brain Tumor Registry of the United States (CBTRUS, 2004–2020) and used to calculate average age-adjusted annual incidence rates (AAAIRs) and incidence rate ratios (IRRs). Poisson regression was used to evaluate associations of VS risk with sex, race/ethnicity, and their interaction (sex*race/ethnicity), both overall and in ten-year intervals of age.

**Results:**

Over an eighteen-year period, 78002 unique individuals received a new diagnosis of VS (52.9% female). Females were at elevated risk compared to males from ages 10–59, after which this trend inverted with males at increased risk. Compared to non-Hispanic White individuals, Hispanic and non-Hispanic Black individuals were at significantly reduced risk of VS throughout the lifespan. The protective effect of non-Hispanic Black race/ethnicity was apparent among both females (IRR=0.40; 95% CI: 0.38–0.42) and males (IRR=0.36; 95% CI: 0.34–0.38), but was significantly stronger among males (P_interaction_<0.001).

**Conclusions:**

Age significantly modifies the relationship between sex and risk of VS, while sex significantly modifies the relationship between race/ethnicity and risk of VS. Findings underscore the importance of incorporating demographic data into studies of VS biology, diagnosis, and clinical management.

## INTRODUCTION

Vestibular schwannoma (VS) is an intracranial peripheral nervous system tumor arising from Schwann cells of the vestibulocochlear nerve division.[1, 2] As the most common non-malignant nerve sheath tumor, it accounts for 6–8% of all primary brain and other central nervous system (CNS) tumor diagnoses in the United States (U.S.).[3] VS incidence increases with age, peaking around the seventh decade of life.[4] Additional demographic risk factors include higher incidence among non-Hispanics than Hispanics, and among non-Hispanic Whites than non-Hispanic Blacks.[4, 5] VS are classified as World Health Organization (WHO) grade 1 tumors and rarely undergo malignant transformation.[6] As a result, VS is frequently diagnosed and monitored radiologically.

Despite being classified as a non-malignant tumor, some VS have been observed to grow rapidly and negatively impact health-related quality of life (HRQOL).[7] Even slow growing lesions, left untreated, can significantly impact quality of life by causing hearing loss, tinnitus, and vestibular dysfunction. Trigeminal neuralgia and facial nerve palsies are also possible, though less common. In the most extreme cases, VS can become life-threatening due to brainstem and cerebellar compression or obstructive hydrocephalus.[2, 8] It remains difficult to forecast tumor growth trajectories or to predict the emergence of debilitating VS-associated neurologic symptoms.[7, 8] Clinical management may include surveillance imaging, stereotactic radiosurgery (SRS), and open microsurgical resection. Use of SRS has increased alongside continued technological improvements, particularly for patients who are not operative candidates. However, microsurgery remains relevant and is especially important for larger tumors and for those refractory to SRS.[9] No one treatment paradigm is definitively superior, and practices vary widely across institutions.

In a previous study of 23,729 new VS diagnoses in data from the Central Brain Tumor Registry of the United States (CBTRUS; 2004–2010), we observed that older age, White race, and non-Hispanic ethnicity were associated with significantly elevated incidence of VS. However, lifetime incidence did not significantly differ between males and females in those data.[4] An updated analysis of CBTRUS data, including 49,869 VS diagnoses (2004–2016), largely recapitulated these results.[5] Additionally, it observed that VS incidence rates declined after 75 years of age and suggested that sex-specific and race/ethnicity-specific risk of VS may be better understood in an age-stratified context. Examining VS incidence rates across strata of age, biological sex, and race/ethnicity may improve our understanding of this common intracranial tumor, potentially enabling earlier detection and more effective interventions. For example, patients diagnosed before age 30 or with bilateral VS may merit clinical workup for an underlying neurofibromatosis Type 2 (NF2) diagnosis.[10]

To address this knowledge gap and comprehensively evaluate potential differences in VS incidence, we analyzed population-based registry data from CBTRUS covering the years 2004–2021. We examined population-level variation in the incidence of non-malignant, intracranial VS over an eighteen-year period, evaluating associations with sex and with race/ethnicity in ten-year intervals across the lifespan. We also explored potential interactions between sex and race/ethnicity, both overall and within each decade of life. Results can help reveal how racial/ethnic differences in VS risk are modified by factors like age and sex, and may have utility in addressing population-level differences in pathways to VS diagnosis and patterns of care.

## METHODS

Study data were obtained from CBTRUS, a population-based registry combining data from the CDC’s National Program of Cancer Registries (NPCR) and NCI’s Surveillance, Epidemiology and End Results (SEER) Program, which covers the entire U.S. population. CBTRUS aggregates primary brain and other CNS tumor incidence data from 52 central cancer registries (48 NPCR and 4 SEER).[3] U.S. central cancer registries must collect data on all cancers diagnosed in the United States in accordance with the Cancer Registries Amendment Act (Public Law 102–15), and on all non-malignant primary tumors of the brain and CNS in accordance with the Benign Brain Tumor Cancer Registries Amendment Act (Public Law 107–260). Cancer registry data are collected, consolidated, and curated by trained staff using information gathered from imaging, pathology and autopsy reports, hospital and physician records, and from outpatient clinic, radiation center, and free-standing imaging center records. Therefore, data from the aforementioned cancer registries include both surgical and non-surgical cases, but do not include occult brain tumors that remain undiagnosed.

Population data from the US Census Bureau were obtained from the SEER program (http://seer.cancer.gov) and used to calculate incidence rates of VS per 100,000 persons, age-adjusted to the 2000 US standard population. We calculated the average annual age-adjusted incidence rate (AAAIR) of non-malignant VS (International Classifications of Diseases for Oncology, 3^rd^ edition [ICD-O-3] morphology code 9560/1) of the acoustic nerve (ICD-O-3 topography code C72.4) in CBTRUS data from 2004 (when reporting of non-malignant CNS tumors became mandatory) through 2021. Tumors diagnosed solely by radiologic means and those confirmed microscopically were both included, but we excluded cases diagnosed by autopsy or death certificate only.[11] The incidence of bilateral VS was not specifically assessed, but has been observed to represent fewer than 1% of all VS diagnoses in CBTRUS.[5] Data regarding the presence of inherited genetic syndromes were not available.

Demographic information was obtained from the CBTRUS database and used to calculate AAAIRs and 95% confidence intervals (CIs) within strata of age, sex, and combined race/ethnicity. AAAIRs were used to calculate incidence rate ratios (IRRs) and 95% CIs for females (versus males) and for both Hispanic individuals and for non-Hispanic Black individuals (versus non-Hispanic White individuals) overall, within 10-year strata of age at diagnosis, and by diagnostic confirmation (radiologic or microscopic).[12, 13] IRRs were considered statistically significant at a P-value <0.05, without correction for multiple testing. Interaction analyses (sex*race/ethnicity) were performed in R 4.4.2 using Poisson models, within 10-year age groups and overall (adjusting for age). Figures were created in R 4.4.2.

## RESULTS

**Fig. 1:**
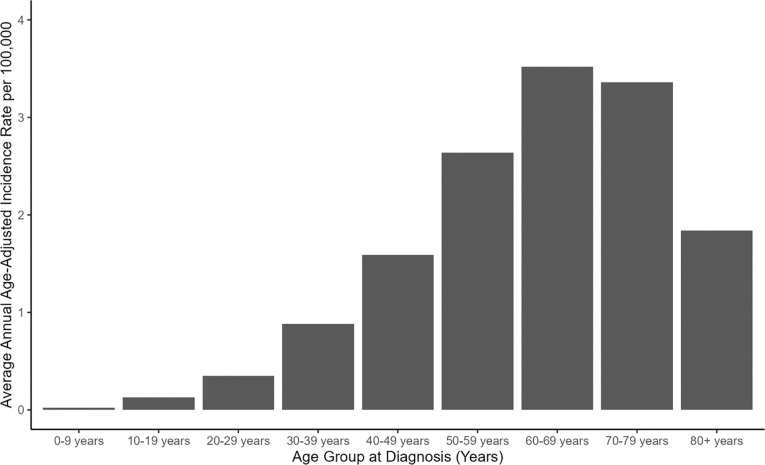
Average annual age-adjusted incidence rates for vestibular schwannoma by 10-year age group at diagnosis. (CBTRUS: Data provided by CDC’s National Program of Cancer Registries and NCI’s Surveillance, Epidemiology and End Results Program, 2004–2021)

**Fig. 2:**
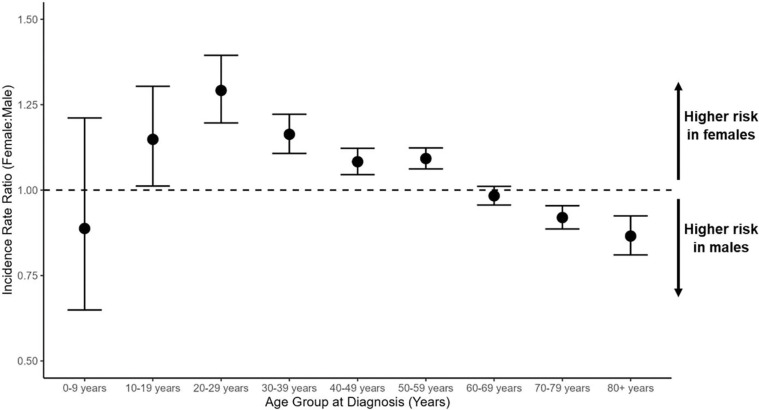
Female-to-male incidence rate ratios (IRR) and 95% confidence intervals (CI) for vestibular schwannoma by 10-year age group at diagnosis. (CBTRUS: Data provided by CDC’s National Program of Cancer Registries and NCI’s Surveillance, Epidemiology and End Results Program, 2004–2021)

Between 2004 and 2021, a total of 78,002 unique individuals were recorded in CBTRUS data as having received a VS diagnosis, including 36,718 males and 41,284 females. 56.6% of cases were diagnosed solely by radiologic means, while 41.2% were microscopically confirmed. The AAAIR of VS increased with age, peaking during the seventh decade of life (*i.e.*, age 60–69) among both males and among females (Figure 1, Table 1). Beginning during the second decade of life, VS incidence was significantly elevated among females compared to males (IRR=1.15; 95% CI: 1.01–1.30; p=0.032) (Figure 2, Table 1). The risk of VS associated with female sex peaked during the third decade of life (IRR=1.29; 95% CI: 1.20–1.39; p<0.0001) but remained significantly elevated through the sixth decade of life (IRR=1.09; 95% CI: 1.06–1.12; p<0.0001). An inverse association emerged in the 60–69 year age group, with women at non-significantly reduced risk of VS compared to men. Female sex was associated with a significant reduction in VS risk among those 70–79 years of age, and female sex remained significantly protective among those ages 80+ (IRR=0.87; 95% CI: 0.81–0.92; p<0.0001). Overall, results indicate that females are at elevated risk of VS compared with males from adolescence until the sixth decade of life, with males at higher risk than females beyond sixty years of age. These patterns remained in regression models adjusted for race/ethnicity (**Supplementary Table 1**), and when analyses were restricted to microscopically confirmed VS (**Supplementary Table 2**) or radiologically confirmed VS (**Supplementary Table 3**).

**Fig. 3:**
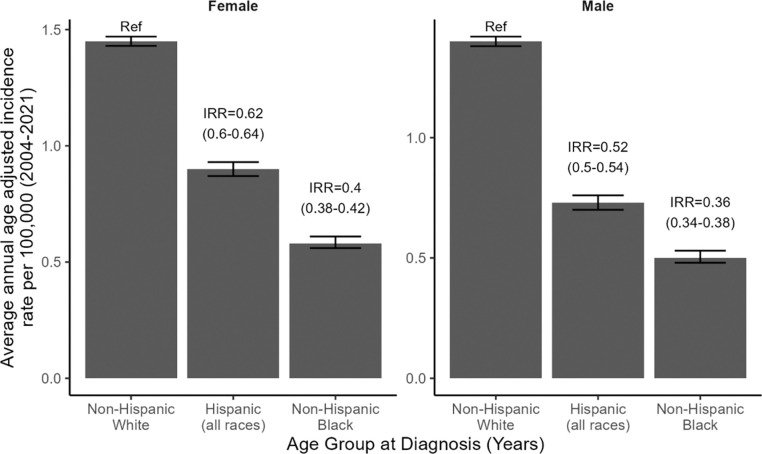
Average annual age-adjusted incidence rate (AAAIR) and 95% confidence interval (CI) for vestibular schwannoma by race/ethnicity and stratified by sex. Rates are age-adjusted to the 2000 U.S. standard population. Incidence rate ratios (IRR) and their 95% CI appear above bars and are calculated relative to non-Hispanic White individuals as the reference. (CBTRUS: Data provided by CDC’s National Program of Cancer Registries and NCI’s Surveillance, Epidemiology and End Results Program, 2004–2021)

We next evaluated VS incidence across strata of race/ethnicity, separately among females and among males. The AAAIR was highest among non-Hispanic White females, with Hispanic females (IRR=0.62; 95% CI: 0.60–0.64; p<0.0001) and non-Hispanic Black females (IRR=0.40; 95% CI: 0.38–0.42; p<0.0001) experiencing significantly lower risk of VS across the lifespan (Figure 3). Results among males were similar, although effect sizes appeared magnified. Specifically, Hispanic males (IRR=0.52; 95% CI: 0.50–0.54; p<0.0001) and non-Hispanic Black males (IRR=0.36; 95% CI: 0.34–0.38; p<0.0001) were at significantly lower risk of VS across the lifespan relative to non-Hispanic White males (Figure 3).

**Fig. 4:**
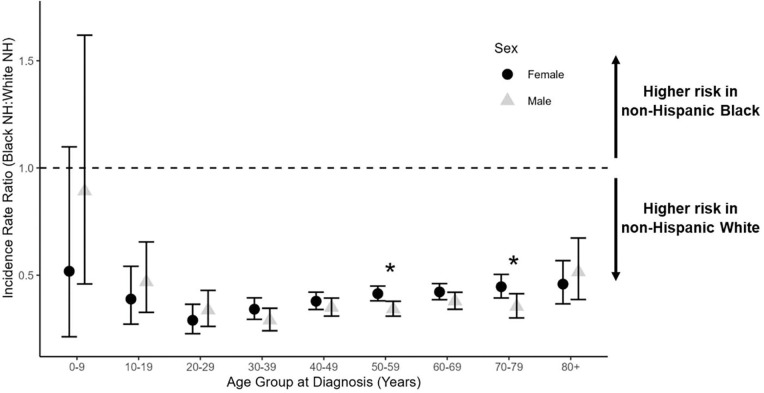
Non-Hispanic Black (NHB) to non-Hispanic White (NHW) incidence rate ratios (IRR) and 95% confidence intervals for vestibular schwannoma by 10-year age group at diagnosis and stratified by sex. Differences between the NHB:NHW IRR in females (black circles) and in males (gray triangles) indicate that sex modifies the relationship between race/ethnicity and VS risk. Significant sex*race/ethnicity interactions are denoted by an asterisk. (CBTRUS: Data provided by CDC’s National Program of Cancer Registries and NCI’s Surveillance, Epidemiology, and End Results Program, 2004–2021)

To further explore the strong protective effect of non-Hispanic Black versus non-Hispanic White race/ethnicity, we calculated IRRs within 10-year intervals of age at diagnosis and across strata of sex. Non-Hispanic Black individuals experienced lower incidence of VS than non-Hispanic White individuals in all age groups, which was true among both females and among males (Figure 4). A significant protective effect of non-Hispanic Black race/ethnicity was observed from the second decade of life onward, which was most pronounced in young adults ages 20–29 years (IRR=0.29; 95% CI: 0.23–0.36; p<0.001) (Figure 4, **Supplementary Table 1**).

Analysis of the interaction between sex and race/ethnicity revealed that the protective effect of non-Hispanic Black (versus non-Hispanic White) race/ethnicity was significantly stronger among males than among females across the lifespan (p<0.001), with interaction terms remaining statistically significant in sensitivity analyses limited to tumors with microscopic (p=0.023) or radiologic (p<0.001) confirmation (**Supplementary Table 4**). Within 10-year age intervals, sex significantly modified the relationship between race/ethnicity and VS risk in the sixth and eighth decades of life (p=0.0030 and 0.014, respectively) (Figure 4, **Supplementary Table 1**). The magnitude and direction of these age-stratified interactions were consistent when stratifying by tumor confirmation status, although sample sizes were reduced and p-values for interaction terms were statistically significant in only the radiologically-confirmed subset of cases (**Supplementary Tables 2–3**). As displayed in Figure 4, the non-Hispanic Black to non-Hispanic White IRR among males was smaller (*i.e.*, more protective) than that among females from ages 30–79. This is a period of time during which >90% of all VS diagnoses in CBTRUS data occurred, which likely accounts for the significant interaction between sex and race/ethnicity across the lifespan despite age-stratified analyses only revealing statistically significant interaction terms within two ten-year windows of age. Thus, a strong protective effect of non-Hispanic Black versus non-Hispanic White race/ethnicity is observed in both sexes, from age 10 onward. Furthermore, this effect appears to be strongest among adult males, despite some variability during very early and very late life when VS diagnoses are substantially less common.

## DISCUSSION

We present eighteen years of nationally representative brain tumor registry data, covering essentially the entire U.S. population. Analyses reveal how age, sex, and race/ethnicity jointly influence VS risk across the lifespan. While lifetime incidence of VS was modestly higher in females than in males, age-stratified analyses revealed a more complex pattern of risk associated with biological sex. Females ages 10–19 were at 15% greater risk of a VS diagnosis than males in this age group, which increased to an approximately 30% greater risk among females in the 20–29 year age group. Although VS risk remained elevated in females until the sixth decade of life, males experienced greater risk of VS beyond 60 years of age, with males at 15% greater risk of a VS diagnosis than females among the oldest Americans (80+ years). We observe significantly elevated VS risk among non-Hispanic White individuals relative to both Hispanic and non-Hispanic Black individuals, and further present age-stratified analyses and explore interactions between sex and race/ethnicity. Results demonstrate that the protective effect of non-Hispanic Black versus non-Hispanic White race/ethnicity is present throughout the lifespan and is significantly more protective among males than among females.

This study expands upon prior epidemiologic analyses of VS, including 2015 and 2020 publications from CBTRUS investigators that captured 23,000 and 50,000 VS diagnoses, respectively.[4, 5] Those studies observed similar patterns as those reported here, in that VS incidence increased with age and was highest among individuals who are non-Hispanic White. Compared with these earlier reports, this new analysis of 78,000 VS diagnoses reveals that VS incidence rates decline after age 70. Leveraging this extremely large sample of VS patients, we also clarify that the effect of sex on VS risk is highly age-dependent, and observe that sex modifies relationships between race/ethnicity and VS risk.

Our results are particularly interesting in the context of the descriptive epidemiology of other primary brain tumors. Females are at elevated risk of meningioma, the most common primary brain tumor, beginning around puberty and continuing throughout their lives. By the fifth decade of life, women are at three-fold higher risk of developing a meningioma compared to men, after which the risk associated with female sex steadily declines.[11] Although we observed a similar pattern in analyses of VS incidence, men were at elevated risk of VS compared to women after age sixty, whereas women remain at elevated risk of meningioma throughout later life. These patterns are also notably distinct from those observed in glioblastoma, the most common primary malignant brain tumor, where males are at elevated risk compared to females throughout the lifespan.[14] On the other hand, the risk of both VS and of glioblastoma are significantly lower in Hispanics and in African Americans compared to non-Hispanic Whites,[15, 16] whereas meningioma risk is significantly elevated in Hispanics and African Americans relative to non-Hispanic Whites.[13, 17]

While this study helps to clarify and expand our understanding of the relationships between demographic factors and VS risk, little is yet known regarding the genetic or environmental etiology of these tumors. As with other primary brain tumors, an elevated risk of VS has been observed in association with exposure to ionizing radiation.[18] Risk of both VS and of meningioma are also significantly elevated in individuals with NF2,[19] and a reduced risk of both VS and of meningioma have been reported among smokers.[20, 21] Hormonal abrogation has been offered as a hypothesized explanation for the inverse associations between smoking and risk of both VS and of meningioma, particularly among females.[22] While a substantial role for sex hormones in the development of meningioma is increasingly accepted, hormonal contributions to the development of VS remains underexplored. A large, prospective study in the UK General Practice Research Database identified a significantly increased risk of VS among women receiving menopausal hormone therapy.[23] While VS tumor cells are not typically observed to express estrogen receptors, a recent study observed progesterone receptor expression in ~20% of tumors.[24] Given age-dependent associations with sex observed in our analyses, where female sex conferred increased risk of VS from ages 10–59 years, the role of sex hormones in VS development likely merits further attention.

Our study has several important limitations. First, VS patients include both those with microscopic confirmation of their diagnosis and those with only radiologic confirmation. However, we conducted sensitivity analyses stratified by diagnostic confirmation method and observed that associations were largely comparable across strata. Completeness of data collection for radiologic diagnoses of non-malignant brain tumors may vary by state, and could potentially contribute to underreporting in specific geographic regions. While the registry-based capture of cases enabled analysis of an extremely large population-based sample of VS patients, it also restricted our ability to adjust for potentially relevant patient-level risk factors, such as smoking status, prior exposure to therapeutic radiation, and NF2 diagnoses. Additionally, because race/ethnicity are correlated with both socioeconomic status and healthcare access, results may be impacted by detection biases due to underdiagnosis in racial/ethnic minority populations and/or overdiagnosis among non-Hispanic White populations. While this a known limitation of registry-based data analysis, such factors could be expected to influence time to diagnosis more strongly than total diagnoses, but the effects of race/ethnicity were extremely consistent across strata of age. Although CBTRUS data cover the entire U.S. population, associations observed in this study may vary globally due to differences in the distribution of demographic and environmental risk factors. The analytic strategy, variable groupings, and interaction terms used here were selected *a priori* to align with our prior publications on meningioma,[11, 13] and it is also possible that alternative approaches (*e.g.*, different groupings of age) may be more optimal for analysis of VS incidence.

While small to medium-sized VS may be followed radiologically without immediate intervention, such tumors—even when not growing—may still present serious neurological complications such as hearing loss, tinnitus, facial palsy, and vertigo.[7, 25] Increasingly, front-line treatment of tumors <2.5cm includes SRS (*e.g.*, Gamma Knife therapy), particularly for older patients, those who are otherwise poor surgical candidates, and those who strongly dislike the idea of surgery.[26] Contemporary studies report tumor control rates around 90%, with relatively little treatment morbidity. However, SRS does not render radiographic cure; tumors may persist indefinitely, and they may even enlarge in the first three years after treatment. Patients who fail to respond to SRS may subsequently undergo salvage microsurgical resection, but this treatment procedure becomes more challenging when navigating through radiated tissue. On the other hand, microsurgical resection remains a front-line option for younger patients, those with larger (especially >3 cm) or more rapidly growing tumors, and those with severe symptoms.[27] Though surgical resection confers immediate reduction of mass effect and often total cure, it is accompanied by the attendant risks of working in the cerebellopontine angle, such as facial neve palsy and cerebrospinal fluid leak. Adjuvant SRS may be employed to treat subtotally resected tumors and recurrent tumors.[26]

Neuro-oncologists and neurosurgeons currently lack robust methods to predict the clinical trajectory of newly-diagnosed VS. However, emerging epigenomic and transcriptomic data suggest that at least two molecular subgroups of VS exist, and that these subgroups may have differing propensities to recur.[28] Although the population-based analyses presented here evaluated demographic factors associated with VS incidence and not with clinical outcomes, the major contributions of sex, age, and race/ethnicity that we observe suggest that these factors should be considered when constructing any model intended to predict VS tumor behavior. Further research is needed to translate findings into cohorts of molecularly profiled VS and to explore the role of both hormones and environmental exposures in contributing to VS risk within and across populations.

## Supplementary Material

Supplementary Files

This is a list of supplementary files associated with this preprint. Click to download.

• VSSupplementalTables.docx

## Figures and Tables

**Table 1.** Total cases, average annual age-adjusted incidence rates (AAAIR), incidence rate ratios (IRR; Ref, Reference), and 95% confidence intervals (CI) for vestibular schwannoma, by 10-year age intervals. (CBTRUS: Data provided by CDC’s National Program of Cancer Registries and NCI’s Surveillance, Epidemiology and End Results Program, 2004–2021).

Age group	Sex	Total Cases (2004–2021)	AAAIR (95% CI)	IRR (95% CI)	P-value
0–9 years	Male	93	0.03 (0.02–0.03)	Ref	
	Female	79	0.02 (0.02–0.03)	0.89 (0.65–1.21)	0.48
10–19 years	Male	473	0.12 (0.11–0.13)	Ref	
	Female	518	0.14 (0.13–0.15)	1.15 (1.01–1.30)	0.032
20–29 years	Male	1,216	0.31 (0.29–0.32)	Ref	
	Female	1,512	0.40 (0.38–0.42)	1.29 (1.20–1.39)	<0.0001
30–39 years	Male	3,010	0.81 (0.78–0.84)	Ref	
	Female	3,478	0.94 (0.91–0.98)	1.16 (1.11–1.22)	<0.0001
40–49 years	Male	5,893	1.52 (1.48–1.56)	Ref	
	Female	6,440	1.65 (1.61–1.69)	1.08 (1.05–1.12)	<0.0001
50–59 years	Male	9,415	2.52 (2.47–2.57)	Ref	
	Female	10,676	2.75 (2.7–2.81)	1.09 (1.06–1.12)	<0.0001
60–69 years	Male	9,632	3.55 (3.48–3.62)	Ref	
	Female	10,356	3.49 (3.42–3.56)	0.98 (0.96–1.01)	0.24
70–79 years	Male	5,429	3.52 (3.42–3.61)	Ref	
	Female	6,037	3.23 (3.15–3.32)	0.92 (0.89–0.95)	<0.0001
80+ years	Male	1,557	2.01 (1.91–2.11)	Ref	
	Female	2,188	1.74 (1.67–1.82)	0.87 (0.81–0.92)	<0.0001

## Data Availability

The National Program of Cancer Registries data used in this request are provided under contract with the Centers for Disease Control. Surveillance, Epidemiology, and End Results Program data are publicly available and can be requested from https://seer.cancer.gov/.
